# Omega-3 mechanism of action in inflammation and endoplasmic reticulum stress in mononuclear cells from overweight non-alcoholic fatty liver disease participants: study protocol for the “Brazilian Omega Study” (BROS)—a randomized controlled trial

**DOI:** 10.1186/s13063-021-05702-x

**Published:** 2021-12-18

**Authors:** Ellencristina Silva Batista, Thaiane da Silva Rios, Vitor Rosetto Muñoz, Joyce Santos Jesus, Marcel Monteiro Vasconcelos, Diogo Thimóteo da Cunha, Jose Luis Marques-Rocha, Susana Castelo Branco Ramos Nakandakari, Roberta Lara, Adelino Sanchez Ramos da Silva, José Rodrigo Pauli, Eduardo Rochete Ropelle, Rania Angelina Mekary, Leandro Pereira de Moura, Enilton Aparecido Camargo, Dennys Esper Cintra

**Affiliations:** 1 Graduate Program of Health Sciences (PPGCS), Federal University of Sergipe, Aracaju, Sergipe Brazil; 2 Laboratory of Nutritional Genomics, School of Applied Sciences, University of Campinas, Pedro Zaccaria, 1300 Zip, Limeira, 13484-350 Brazil; 3 Nutrition Department, Federal University of Sergipe, Lagarto, Sergipe Brazil; 4 Lipids and Nutrigenomics Research Center, School of Applied Sciences, University of Campinas, Limeira, Brazil; 5 Laboratory of Molecular Biology of Exercise, School of Applied Sciences, University of Campinas, Limeira, Brazil; 6 Multidisciplinary Laboratory of Food and Health, School of Applied Sciences, University of Campinas, Limeira, Brazil; 7 Department of Integrated Health Education, Federal University of Espírito Santo, Vitoria, Brazil; 8 School of Physical Education and Sport of Ribeirão Preto, University of São Paulo (USP), Ribeirão Preto, São Paulo, Brazil; 9 Massachusetts College of Pharmacy and Health Sciences (MCPHS) University, Boston, MA USA; 10 Department of Neurosurgery, Brigham and Women’s Hospital, Harvard Medical School, Boston, MA USA; 11 Department of Physiology, Federal University of Sergipe, São Cristóvão, Sergipe Brazil

**Keywords:** Overweight, GPR120, Omega-3, Inflammation, Endoplasmic reticulum stress, NAFLD

## Abstract

**Abstract:**

The low-grade inflammation is pivotal in obesity and its comorbidities; however, the inflammatory proteins are out of target for traditional drug therapy. Omega-3 (ω3) fatty acids can modulate the downstream signaling of Toll-like receptor (TLR) and tumor necrosis factor-α receptor (TNFα) through GPR120, a G-protein-coupled receptor, a mechanism not yet elucidated in humans. This work aims to investigate if the ω3 supplementation, at a feasible level below the previously recommended level in the literature, is enough to disrupt the inflammation and endoplasmic reticulum stress (ER-stress), and also if in acute treatment (3 h) ω3 can activate the GPR120 in peripheral blood mononuclear cells (PBMC) and leukocytes from overweight non-alcoholic fatty liver disease (NAFLD) participants. The R270H variant of the *Ffar4* (GPR120 gene) will also be explored about molecular responses and blood lipid profiles. A triple-blind, prospective clinical trial will be conducted in overweight men and women, aged 19–75 years, randomized into placebo or supplemented (2.2 g of ω3 [EPA+DHA]) groups for 28 days. For sample calculation, it was considered the variation of TNFα protein and a 40% dropout rate, obtaining 22 individuals in each group. Volunteers will be recruited among patients with NAFLD diagnosis. Anthropometric parameters, food intake, physical activity, total serum lipids, complete fatty acid blood profile, and glycemia will be evaluated pre- and post-supplementation. In the PBMC and neutrophils, the protein content and gene expression of markers related to inflammation (TNFα, MCP1, IL1β, IL6, IL10, JNK, and TAK1), ER-stress (ATF1, ATF6, IRE1, XBP1, CHOP, eIF2α, eIF4, HSP), and ω3 pathway (GPR120, β-arrestin2, Tab1/2, and TAK1) will be evaluated using Western blot and RT-qPCR. Participants will be genotyped for the R270H (rs116454156) variant using the TaqMan assay. It is hypothesized that attenuation of inflammation and ER-stress signaling pathways in overweight and NAFLD participants will be achieved through ω3 supplementation through binding to the GPR120 receptor.

**Trial registration:**

ClinicalTrials.gov #RBR-7x8tbx. Registered on May 10, 2018, with the Brazilian Registry of Clinical Trials.

**Supplementary Information:**

The online version contains supplementary material available at 10.1186/s13063-021-05702-x.

## Background

The world is losing the war against obesity [1], a phenomenon endorsed by ineffective therapeutic drugs or unsuccessful weight loss maintenance after bariatric surgery [2]. It is well recognized that excess saturated fatty acids from ultra-processed foods, de novo lipogenesis, and visceral adipose tissue efflux can activate and intensify pro-inflammatory signaling and endoplasmic reticulum stress response (ER-stress) while reducing systemic insulin sensitivity [3–6]. The hyperglycemia-induced by impaired glucose uptake by the skeletal muscle and increased hepatic gluconeogenesis and glycogenolysis [5–9] lead to non-alcoholic fatty liver disease (NAFLD) development, common obesity-associated comorbidity [6].

Monocytes and lymphocytes play a pivotal role in inflammation and may be actively involved in NAFLD development or progression [10, 11]. Leukocytes are sensitized by inflammatory proteins and respond to changes in the levels of the fatty acid by regulating a wide gene network, including those involved in immune response and lipid metabolism [12]. Because NAFLD is directly associated with increased plasma lipid levels, it is possible that data obtained from isolated peripheral blood mononuclear cells (PBMCs) could reflect the in vivo condition and contribute to biological and clinical insights into the human NAFLD process [10, 11]. ER-stress and activation of the unfolded protein response (UPR) have been linked to a variety of inflammatory and stress signaling systems mainly mediated by c-JUN N-terminal-Kinase (JNK) and inositol-requiring enzyme 1α (IRE1α) proteins. Inflammatory and ER-stress signaling in PBMC seems to reflect changes occurring in metabolically active organs such as the adipose tissue, skeletal muscle, and liver [10, 11].

During obesogenesis, the liver can be affected by inflammation triggered by TLR and sustained by cytokines and its receptors such as tumor necrosis factor-alpha (TNFα), interleukins (IL) 1, and IL6 [13–15]. Both intracellular pathways converge to the phosphorylation of TAK1 (TGFβ-activated kinase1), which activates the canonical NFκB pathway [16–18]. Thus, the non-pharmacological approach of omega-3 (ω3) fatty acids emerges as a potential strategy to control the systemic pro-inflammatory tonus once ω3 attenuates the TAK1 activity [4, 17, 19–22].

Since the 1970s, ω3 has been used and evidenced as an anti-inflammatory due to changes in the ω6: ω3 ratio [23, 24]. However, during the 2000s decade (2000–2010), some G-protein-coupled receptor (GPCR) members were identified as possible fatty acids’ receptors. Among them, GPR120 was recognized as ω3 being its major activator [25]. Then, in 2010, a hallmark work of Oh et al. [17] showed the detailed molecular mechanism of ω3 as an anti-inflammatory nutrient. On the cell surface, ω3 fatty acids (eicosapentaenoic—EPA-[C20:5] or docosahexaenoic—DHA-[C22:6]) bind to GPR120, while attracting the intracellular beta-arrestin 2 (βarr2) protein and inhibiting TAK1 phosphorylation in hepatocytes and adipocytes. This mechanism appears to be reinforced when βarr2 also recruits the NLPR3 (NOD-, LRR-, and pyrin domain-containing protein 3) protein, disrupting the inflammasome, a structure involved in the IL1β and IL18 maturation [26]. After GPR120/ω3 internalization induced by βarr2, free ω3 in the cytosol can be derived into pro-resolutive mediators such as resolvins, maresins, and protectins, which are well recognized ω3 subcompounds [27, 28]. The dynamic antiinflammatory pleiotropism orchestrated by ω3 is strengthened when this molecule activates the peroxisome proliferator-activated receptor-gamma (PPARγ), in which it blocks the nuclear translocation of NFkB [18].

Nonetheless, some clinical trials were not able to show any ω3 benefits in human health [29, 30]. Some guidelines for the management of cardiovascular diseases demonstrate weak evidence or do not recommend ω3 fatty acids as weak evidence or without the strength of recommendation [31, 32]. Therefore, we propose an investigation to understand the reason for this “conundrum” in ω3 knowledge. The present study aims to clarify the recognized but not solved biases, commonly found in studies such as [1] uncertified ω3 fatty acid content in capsules or fish’s flesh, [33] [2] uncontrolled bioavailability from gut to the bloodstream and bioaccessibility from the blood to cell incorporation/uptake, [3] ω3 discrepant doses [4], [4] ω3 EPA: DHA blend, [5] incorrect placebo choices [34, 35], and [6] the presence of GPR120 polymorphism [36].

Here, overweight/obese NAFLD patients are chosen in such a way to deviate from cardiovascular studies’ possible biases. Thus, they will be monitored on their inflammatory and ER-stress pathways in PBMCs and neutrophils.

## Subjects and methods

### Study design and settings

This is a triple-blinded (investigators, statistical analysts, and participants), single-centered, randomized placebo-controlled, prospective intervention trial. This protocol is written following the Standard Protocol Items: Recommendations for Interventional Trials (SPIRIT) checklist [37]. The study is designed to investigate the molecular action mechanisms of ω3 as antiinflammatory and anti-ER stress through GPR120 receptor in peripheral blood mononuclear cells (PBMC) and leukocytes of overweight and obese subjects with visceral obesity and non-alcoholic fatty liver disease (NAFLD). The study protocol was approved by the Ethics Committee of the Federal University of Sergipe (#80476517.2.0000.5546) and registered at the Brazilian Registry of Clinical Trials (#RBR-7x8tbx), on May 10, 2018, retrospectively registered: https://ensaiosclinicos.gov.br/rg/RBR-7x8tbx. All outpatients will be informed by study design before signing the informed consent form.

In the first part of the intervention (acute measurement [3 h after the first capsule intake]), the surrogate outcomes will be [1] ω3 fatty acid concentration in bloodstream, [2] association between GPR120 and βarr2 proteins, and [3] ω3 fatty acid concentration into PBMCs. In the second part, after chronic treatment (28 days of supplementation), the primary outcome will be considered the reduction (upper to 10%) in the TNFα and IL6 protein contents in PBMCs. Both parts of the intervention will be carried out in PBMC and leukocytes. Outcomes such as body weight, waist circumference, and glycemia will not be considered due to short supplementation time and study aim.

### Eligibility

Eligibility criteria will be overweight or obese subjects (BMI > 24.9 kg/m [2]), with visceral adiposity (waist circumference > 80 cm for women and > 94 cm for men) [38], the age range of 19–75 years. Outpatients, who were for the first time consulted by an expert physician, from January 2016 to December 2018, at the Hepatology Clinic of the Federal University of Sergipe, Aracaju, Brazil, were diagnosed with non-alcoholic fatty liver disease (NAFLD) using ultrasound and laboratory tests. The exclusion criteria will consider any history of chronic liver diseases, such as viral or drug hepatitis, confirmed Wilson disease, primary hemochromatosis, suffering from hyper or hypothyroidism, cancers, diabetes *mellitus* type 1, diabetes mellitus type 2 in insulin therapy, obesity due to excessive use of corticosteroids, Cushing’s syndrome, patients with chronic infections, autoimmune diseases, in abuse of alcohol, in use of hepatotoxic drugs, pregnant, and breastfeeding women. Additionally, patients who are not able to participate in the standardized anthropometric evaluation in the study, such as patients with amputated limbs and those who cannot answer the questionnaires, such as the ones with neurological diseases, will not be eligible. Patients with acute myocardial infarction and stroke in the last 5 years are precluded from participating. Individuals taking anti-inflammatory drugs and who in the last 3 months before the study made use of nutritional supplements containing oils, fats, or fatty acids will be excluded. Those who had a weight loss of more than 10% of body weight in the last 3 months will also be excluded.

### Sample size calculation

The sample size calculation was carried out using the G * Power 3.1.9.2 software, and the following values and information were considered: [1] two groups (control and treatment) and [2] effect size *d* = 1.15 considering the variation of TNFα/βactin protein observed in the study by Huang et al. (2016) [39]. In this study, the control group showed 0.45±0.04 (average of arbitrary units ± standard deviation) for TNFα while the telmisartan treated group showed 0.36±0.04 [39]. As in the reference study [39], three types of drugs were used (telmisartan, cyclosporine, and 4-aminopyridine), and the least effective was chosen, understanding that eating will have a possible minor effect concerning hypertensive drugs. This procedure may reduce type II error, [3] type I error *α* = 0.05, and [4] sampling power (1 - *β*) = 85% [5]. The sample size needed was 16 individuals in each group. However, considering a 40% dropout rate, it will be selected 22 per group.

### Recruitment

Patients’ volunteers will be recruited from the Hepatology Clinic via a phone call to interview and screen participants according to inclusion and exclusion criteria. For each participant, there will be a maximum of 10 attempts to make calls upon the first contact asking participants to fill the questionnaire; if he/she cannot fill the questionnaire, another ten attempts to conduct the phone interview will be made. For a participant to be eligible, one has to agree to answer a screening questionnaire based on the Surveillance of Risk Factors and Protection Survey for Chronic Non-Communicable Diseases by Telephone Interviews (Vigitel) [40]. Then, another interview will be scheduled to confirm the data obtained by phone and the nutritionist will explain the study procedures and consent form to the participants. Participants who agree to participate in the study will sign the consent form and go through the initial assessment. Then, 44 individuals will be randomly drawn for participation in the study, and subsequently, they will be randomized into the study groups: placebo and ω3. To maintain confidentiality, each participant will be codified with a number, and only one co-author of the study will have the complete name and data of the participants.

### Assessments to randomization

Before the randomization, an assessment will be made, including a questionnaire about sociodemographic data, alcoholism, smoking, prescription drugs, anthropometric assessment, and body composition (through bioelectric impedance). A blood sample collection will be performed for assessment of plasma glucose levels, insulin, ferritin, glycated hemoglobin, total cholesterol, HDLc and LDLc lipoproteins, triacylglycerol, alanine aminotransferase, aspartate aminotransferase, and C-reactive protein at the Hepatology Clinic.

### Randomization

The distribution between groups, placebo or ω3, will be randomly assigned into groups, placebo or ω3. A sequence number will be generated by SPSS 25.0 under the ratio of 1:1. The numbers will be placed in a sealed envelope and randomly distributed to the participants. After, participants will be listed in ascending order based on biochemical and anthropometric variables, grouped in blocks of two, and then allocated to each of the groups in an equalized manner to ensure group homogeneity. All eligible individuals by the users of the selection will receive a unique and non-transferable code. An independent data manager, who is not involved in clinical practice or patient recruitment, will create the randomization sequence.

### Interventions

#### Omega-3 and placebo

The ω3 capsules were donated by Naturalis®, Nutrition and Pharma Company, São Paulo, Brazil. Each capsule contains 1 g of fish oil in the form of triglycerides, which is equivalent to about ≈ 750 mg of ω3 ([EPA–190 mg; DHA–550 mg], Table 1). The quality of the oil was certified by mass spectrometry (as shown by the Omega-3 determination method below). The heavy metals are under a minimum limit allowed daily intake (arsenic [<0.1 ppm], cadmium [<0.01 ppm], lead [<0.05 ppm], and mercury [<0.005 ppm]). Placebo pills consist of 1 g of mineral oil and were donated by the Catalent Pharma, Inc., Company, Sorocaba, Brazil.

**Table 1:** Fatty acid composition of a capsule containing 1 g of pure fish oil

Carbons/insaturations/ω-position	Nomenclature (IUPAC)	mg/g
**C8:0**	Octanoic acid, methyl ester	0.0411
**C10:0**	Decanoic acid, methyl ester	0.0564
**C14:0**	Methyl tetradecanoate	0.3816
**C16:0**	Hexadecanoic acid	1.4015
**C16:1**	Hexadecenoic acid	0.6626
**C18:0**	Methyl stearate	1.8020
**C18:1 (ω9)**	Octadecenoic acid	3.6400
**C18:2 (ω6)**	Octadecadienoic acid	0.3841
**C18:3 (ω3)**	Octadecatrienoic acid	0.1981
**C19:0**	Nonadecanoic acid	0.1619
**C20:1**	cis-11-Eicosenoic acid	3.5847
**C20:1**	cis-13-Eicosenoic acid	0.5901
**C20:2 (ω3)**	8,11-Eicosadienoic acid	0.4160
**C20:2 (ω6)**	11,14-Eicosadienoic acid	0.4051
**C20:3**	Eicosatrienoic acid	0.2284
**C20:4 (ω6)**	Eicosatetraenoic acid	1.6491
**C21:0**	Heneicosanoic acid	0.3023
**C20:5 (ω3)**	Eicosatetraenoic acid	19.0560
**C22:0**	Docosanoic acid	0.8260
**C22:1**	Docosenoic acid	6.3434
**C22:4 (ω6)**	Docosatetraenoic acid	0.3351
**C22:6 (ω3)**	Docosatetraenoic acid	55.0547
**C24:0**	Tetracosenoic acid	2.4781
	∑SAT	7.45
	∑MONO	14.8208
	∑POLY	77.7266
	∑**ω6**	2.7734
	∑**ω**3∗	74.3087

*IUPAC* International Union of Pure and Applied Chemistry, *ω* Omega, *Σ* sum, *SAT* saturated fatty acids, *MONO* monounsaturated fatty acids, *POLY* polyunsaturated fatty acids. *EPA, DHA plus other ω3 species

#### Procedures

**Fig. 1:**
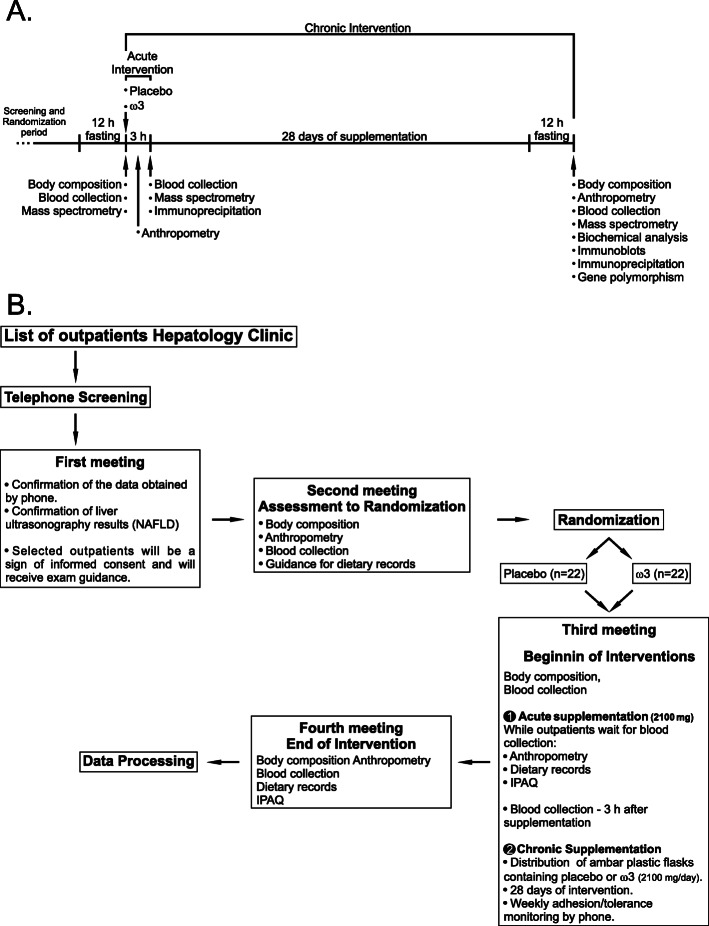
Experimental interventions scheme and detailed subject flow. **A** After the screening and randomization period, the selected participants will be fasting for 12 h. At the hospital service, body composition will be carried out, followed by the blood collection for biochemical and mass spectrometry analysis. Immediately, participants will take their placebo or ω3 capsules and wait for 3 h. Anthropometric measurements will then be performed, the same collections will be repeated, and another part of the blood will be separated to an immunoprecipitation test to verify the association between GPR120 and βarr2 proteins. After 30 days of supplementation, all measurements will be repeated with the inclusion of immunoblot blood tests and polymorphic GPR120 gene analysis. **B** Detailed flow through the study. ω3 Omega-3. IPAQ International Physical Activity Questionnaire

Figure 1A illustrates the simplified acute and chronic intervention design, and Fig. 1B illustrates the overall design and subject flow through the study. Participants may be withdrawn from the study at their request. Upon the first meeting, after being fully informed about the study and all risk factors, patients will be asked to sign the informed consent form. At this moment, the clinical questionnaire will be administrated. Instructions on a blood collection and body bioelectrical impedance test will be given. A second meeting will follow to conduct the randomization of the groups. Body composition and anthropometric profiles will be assessed, and the blood will be collected from all participants. Participants will be instructed to fill three dietary records. After randomization and 12 h of fasting, in the third meeting, the acute supplementation phase will begin. Initially, participants will perform the bioelectrical impedance test before the blood collection and before the ingestion of three capsules of placebo or three capsules of ω3 (totaling 2.2 g of ω3 [EPA–570 mg, DHA–1650 mg]), depending on the group in which they were randomized. In the meantime, because they have to wait for 3 h for the second blood collection to assess the ω3 bioavailability and ability to activate the GPR120 receptor, a 24-h recall (R24h), three dietary records will be administered and physical activity level, and anthropometric profiles will be evaluated. At the end of this meeting, an amber plastic flask protecting the capsules from light and oxygen will be provided to each participant, containing capsules available for 28 days of supplementation (3 caps/day or 2.2 g of ω3). Two days after this last meeting, the patients will report by phone call their R24h. At the last meeting and after 12 h fasting, all these assessments will be repeated.

#### Blinding

The practitioners, participants, outcome assessors, and statisticians will be kept blinded to treatment allocation throughout the trial. No toxic/adverse effects, beyond eructation, have been reported in previous studies using Omega-3 in a similar dose adopted by this study. Capsules will be prepackaged into opaque and identical containers labeled with codes by a local pharmacy, with the investigator’s details and supplementation instructions. This ensures the total blinding of both the participants and investigators. In the event of adverse events, clinicians will be able to unblind participants if they consider this necessary.

#### Safety, compliance monitoring, and participant retention

During follow-up, once a week, participants will report adverse events by phone, which is expected such as eructation, followed by uncomfortable gastric odor. The weekly contact will also promote participant retention to complete the study. In the case of significant adverse effects, the participant will be evaluated by the medical service from the hospital. Together with medical service, the nutritionist leader of this project will be responsible for participant stopping. Treatment compliance will be assessed by the plasma fatty acid content of patients at the beginning and the end of the experiment, which will be analyzed using a gas-chromatograph coupled to a mass spectrometer [41, 42]. See the experimental design of the interventions in Fig. 1 and the detailed schedule of assessments depicted in Table 2.

**Table 2:**
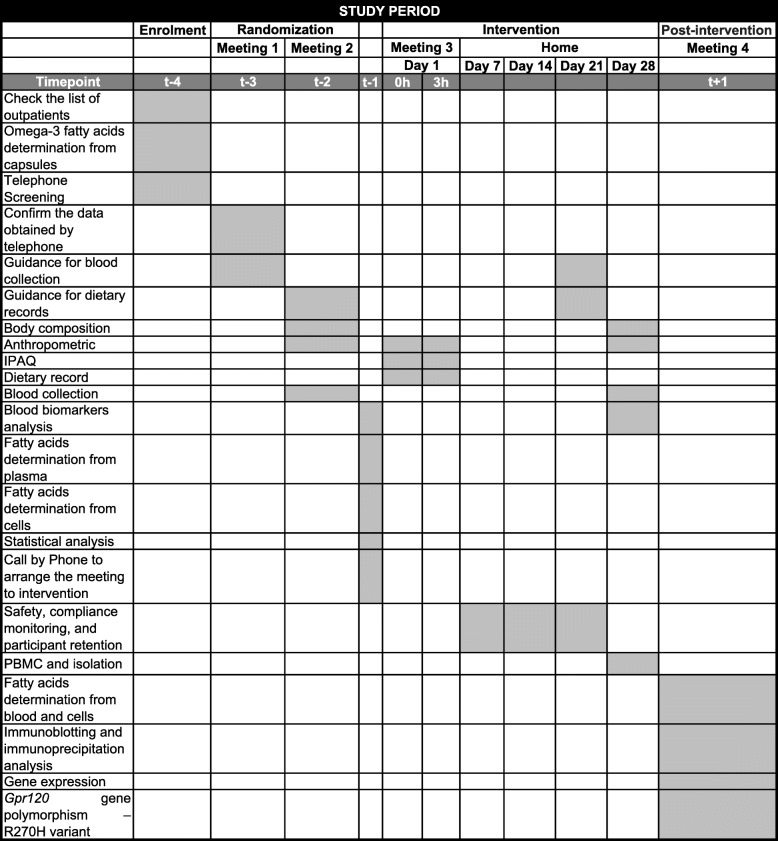
Schematic schedule of enrolment

#### Anthropometric data

Anthropometric (body weight, height, waist-to-height ratio, waist and neck circumferences, and four skin folds) and body composition (electric bioimpedance) data will be assessed at baseline and post-intervention, following respective standardized guidelines and cut-offs [38, 43–46].

#### Food intake

Food intake data will be obtained before and after supplementation using two R24h and three dietary records using the photographic record. Two R24h will be performed on non-consecutive days during the week and one at the weekend. The two R24h will be held on non-consecutive days, one in the third meeting and another after 2 days by phone call. The intake of each food item will be transformed into daily consumption (grams or milliliters). After, total caloric intake, nutrients (carbohydrates, proteins, fats, vitamins, and minerals) and other specific components (fibers, carotenoids, sugars, and fatty acids) will be calculated according to the nutritional composition of each source food provided priority by the Brazilian Food Composition List (TACO) [47] and by the Household Budget Survey data (IBGE, 2010) [48] when food is not found in the first one, with the help of Microsoft Excel (v. 2016) and SPSS (v. 22) software to organize and analyze the data. The values of energy consumption, nutrients, food groups according to nutritional similarity, and food groups according to the degree of industrial processing derived from the four 24hRs will be disattenuated (corrected by intra-individual variability), generating unique values for each item. This will be achieved through the PC-SIDE program (Department of Statistics, Iowa State University, Iowa, USA) developed by the National Research Council and Iowa State University [49]. Moreover, consumption values will be adjusted for energy intake by the residual method [50]. The outpatients will be instructed not to change their dietary patterns.

#### Physical activity

The IPAQ (International Physical Activity Questionnaire) validated for the Brazilian population will be used to assess physical activity levels [51]. After being measured, the outpatients will be instructed not to change their physical activity patterns.

#### Prescribed medications

As described in the eligibility session, only patients under anti-inflammatory therapy will be excluded. There are no restrictions for any other medications; however, any alteration on prescribed medications will need to be immediately communicated.

#### PBMC isolation

After blood collection into heparinized tubes, the samples will be prepared with the use of histopaque-1077 (Sigma, St. Louis, MO, USA). In brief, 15 mL of the blood will be carefully layered onto 15 mL of histopaque. Bands containing the mononuclear cells, leukocytes, and red cells will be collected separately. The PBMCs will be washed with saline, centrifuged, and the supernatant removed [52]. This step will be repeated twice, and three parts of the cell suspension will be destined for immunoblotting/immunoprecipitation, RT-qPCR, and lipid profile analysis. Neutrophils and red blood cells will be lysed using a standard lysis buffer and re-suspended in Trizol® reagent (Life Technologies), storing at −80 °C for RT-qPCR analysis.

#### Immunoblotting and immunoprecipitation analysis

After isolation, PBMCs and neutrophils will be immediately homogenized in solubilization buffer at 4 °C [1% Triton X-100, 100 mmol/L Tris–HCl (pH 7.4), 100 mmol/L sodium pyrophosphate, 100 mmol/L sodium fluoride, 10 mmol/L EDTA, 10 mmol/L sodium orthovanadate, 2.0 mmol/L PMSF, and 0.1 mg aprotinin/mL]. For these cells or plasma proteins, the quantification of total protein will be carried out according to the bicinchoninic acid method [53]. Samples containing 50 μg of protein extracts will be separated by SDS–PAGE, transferred to nitrocellulose membranes, and blotted with antibodies against inflammatory markers: TNFα, MCP1, IL1β, IL6, IL10, JNK, and TAK1; ER-stress markers: ATF1, ATF6, IRE1, XBP1, CHOP, eIF2α, and eIF4; and ω3 pathway markers: GPR120, βarr2, and TAB1/2. In some experiments, the association between GPR120 and βarr2 or βarr2 and TAB1/2 will be evaluated by performing immunoprecipitation. Samples of total protein extracts containing 200 μg of protein will be used for immunoprecipitation with antibodies against GPR120, βarr2, or TAB1/2 and protein G-sepharose at 4 °C overnight, followed by SDS/PAGE and will be transferred to nitrocellulose membranes and blotting with respective antibodies. The bands will be labeled by chemiluminescence and its visualizations performed by GBOX-Chemi-XX6 from Syngene® (Cambridge, Eng).

#### Gene expression

Leukocytes and PBMCs re-suspended in Trizol® reagent (Life Technologies) will be submitted to RNA extraction according to the manufacturer’s instructions. Reverse transcription reaction will be performed using a commercially available set of High Capacity cDNA Archive Kit (Applied Biosystems, USA). The cDNA will be prepared from 1 μg of mRNA, with random hexamer primers, for 10 min at 25°C, 2 h at 37 °C, and 4 °C thereafter on a PCR thermocycler Gene (Applied Biosystems, USA). The resulting cDNA will be diluted to a final concentration of 5 ng/μL to constitute a matrix in further experiments. For real-time quantitative PCR amplification, the TaqMan system (Thermo Scientific®) using specific probes and primers (*Tnfα*, *Il1β, Il6, Il10, Ire1, Perk,* and *Atf6*) gene evaluation will be used, and *Gapdh* will be used as a constitutively expressed gene in these cells. The 7500 real-time qPCR equipment (Applied Biosystems®) will perform fluorescence readings. Expression levels of the target gene transcribed in each sample will be calculated by the comparative Ct method (formula 2^ΔCt^) after normalization with the endogenous gene (*Gapdh*) from Applied Biosystems®.

#### *Gpr120* gene polymorphism—R270H variant

The GPR120 gene variant R270H (rs116454156) polymorphism will be genotyped using the Taqman allelic discrimination technique in the 7500 real-time qPCR equipment (Applied Biosystems®), following the method proposed by Ichimura et al. (2012) [36].

#### Omega-3 fatty acid determination from capsules, blood, and cells

Fifty μL of oil obtained directly from ω3 capsules will be submitted to saponification and esterification following the method of Hartman and Lago [54]. To determine the free fatty acid profile from the blood, 150 μL of the plasma will be directly methylated according to the previously described procedure (Shirai et al., 2005) [55]. Fatty acid methyl esters will be analyzed using a gas chromatograph coupled to the mass spectrometer (model GCMS-QP2010 Ultra; Shimadzu) and a fused-silica capillary Stabilwax column (Restek Corporation, U.S.) with dimensions of 30 m × 0.25 mm internal diameter coated with a 0.25-μm-thick layer of polyethylene glycol. The experimental conditions will be followed by the method described by Cintra et al., 2012 [4].

#### Data treatment and statistical aspects

A steering committee will be organized for trial supervision. The process of data collection and management will be monitored by them. The committee may recommend and request the principal investigator to make some changes to the plan. To increase the security and data quality, the records will be inputted at the bank by one person and checked by another one. Data analysis will be performed according to the intention-to-treat principles [56]. The software Statistical Package for Social Sciences (SPSS), v. 22.0, will be used for data entry and analysis. Statistical analysis will be performed through descriptive analysis, with numerical variables expressed as the mean and standard deviation (SD) or median and interquartile range. All data will be subjected to the Shapiro-Wilk normality test. First, a two-way analysis of variance will be performed to check intra- and intergroup differences. Time will be added as a repeated factor, treatment as an independent factor, and the other variables (e.g., anthropometric data, food intake, blood analysis) as covariates.

The paired Student’s *t* test or Wilcoxon’s test will be used to compare the time effects. The Student’s *t* test and Mann-Whitney *U* test will be used to investigate group differences (placebo and treatment). All tests will be considered significative *P*<0.05. After trial completion, the principal investigator (PI) will be responsible for data archiving and preservation. Only investigators can access data. The PI will be responsible to terminate the trial at any moment.

#### Reporting of the study results

At the end of the experimental period, the results of the biochemical and anthropometric analysis will be delivered to patients in person. Also, at the end of the study, a meeting will be planned to discuss the results individually. Absent patients will receive their summarized results by email.

## Discussion

Due to the multifaceted ω3’s ability to modulate inflammation [17, 22, 26, 28], it is not surprising to find ω3 as a panacea; however, this most widely investigated nutrient still has many unsolved questions. Several non-controlled biases and confounding factors compromise the real interpretation of ω3 actions, even those well controlled and with a high number of participants [57]. To elucidate some points related to ω3 fatty acids in human health, we designed a triple-blinded, randomized placebo-controlled intervention trial. This study has an experimental design, followed by an acute and chronic intervention in overweight and NAFLD participants, with different molecular approaches from other studies. If our strategy works, it can contribute to improving the rationale of new studies considering the solutions proposed ahead. Despite our objectives being related to w3 actions in NAFLD patients, the original studies included in our protocol were selected from cardiovascular outcomes, as this was the most investigated area of study, which contained an appropriate sample size to demonstrate and achieve statistical significance. In addition, many of these studies used similar dosages of ω3 allowing comparisons among studies.

The EPA and DHA are the most studied among ω3 species; however, this remains without any standardization related to its origin, whether from flesh fish or capsules. De Mello et al. (2009) [58] compared the effect of fatty vs lean fish intake on inflammatory markers in patients with coronary heart disease. After 8 weeks, no differences were found between the groups. The authors used six types of fatty fishes (salmon, rainbow trout, Baltic herring, tuna, whitefish, or vendace); however, the fish’s flesh fatty acid profile was not determined before the intervention, and the variation can be high among them. In fishes such as trout (*Oncorhynchus mykiss*) and salmon (*Salmo salar*), DHA is the primary ω3 prevalent type in the whole body, although fatty acids are distributed differently among their tissues. Betancor et al. (2017) [59] tested different diets for salmons and found in the control group (wild type) 6.8% of DHA in the flesh, similar to 6.2% in the flesh of rainbow trout found by Cintra et al. (2006) [60]. Despite being considered as fatty fishes, the lipid depots are out of the muscle, undisputedly the most used part in studies with humans. Betancor et al. (2017) [59] also found 25% of DHA in the liver of salmon, similar to the 27% on trout observed by Satué et al. (1996) [61]. Other conditions hard to control for are the fish gender [61] and stage of sexual maturation [62], both of which influence the fatty acid composition in all of the fish parts.

Considering these difficulties, we will adopt ω3 supplementation by capsules, even though there are several considerations to be noticed. One struggling question among the studies is the lack of oil quality monitoring in capsules [57, 63]. We previously tested some supplement brands, choosing the one with the highest similarity to the natural profile found in wild fishes from cold water. The capsules were submitted to fatty acid profile determination by a gas-chromatography coupled to a mass spectrometer, an approach considered the gold standard, and should be obligatory before interventions due to routine adulteration [63, 64]. In our study, we had access to the pharmaceutical company and traced the capsules, following the encapsulation process, since their origin (including placebo [mineral oil] capsules). The fresh capsules will also be tested a day before the beginning of the study.

Dose determination of ω3 supplement is as important as its quality. No standardized dose has been used in the literature and most of the doses used are considered very high. It is common to find well-controlled trials using ω3 associated or not with beneficial effects, under low or high doses. Considering low ω3 doses, a recently randomized placebo-controlled trial showed 12.933 participants treated with 840 mg/day of ω3 capsules (EPA 460 mg and DHA 380 mg) during 5.3 years, aiming for primary prevention (myocardial infarction, stroke, and cardiovascular mortality) [29]. The authors did not find evidence of the differences in outcomes of major cardiovascular events. On the other hand, the GISSI-Prevenzione Trial tested in 2.836 patients, a very similar ω3 dose (850 mg EPA+DHA/day [1:2]) during 3.5 years, and found a reduced rate of death, non-fatal myocardial infarction, and stroke [65]. When ω3 doses were increased, the same incongruences (major cardiovascular events) were noticed. Sixteen mild-hypertension patients taking two daily ω3 capsules (each capsule containing EPA 460 mg and DHA 380 mg), during 3 months, did not have their blood pressure altered after treatment [66], while another study followed 23 hypertensive patients using 4 g/day ω3 (each capsule containing EPA 465 mg and DHA 375 mg) during 2 months, found a very significant reduction on systolic (*P*<0.03) and diastolic (*P*<0.003) blood pressure [67]. Here, we decided to use 2.2 g of ω3 (3 capsules, 1 g each, containing EPA 190 mg and DHA 550 mg, Table 1), an intermediary ω3 dose found in relevant studies [34, 68].

Still regarding the dosage, the EPA: DHA blend is another non-standard characteristic presenting several divergent results when EPA or DHA is predominant [12, 69, 70, 71]. The difficulty in this interpretation is worsening once a recent study showed the prominent and very consistent reduction in the risk of ischemic events, cardiovascular death, and hypertriglyceridemia on 4.089 patients using 2 g of icosapent ethyl [21]. The icosapent ethyl is a highly purified ω3 from EPA (C20:5), differentiating itself because it contains two more carbons after the ester bond (C22:5). It is also important not to confound it with docosapentaenoic acid (DPA), which has the same number of carbons and double-bounds (C22:5), without natural occurrence [21].

The nutrigenomic approaches have clarified relevant differences between EPA and DHA molecules. During 10 weeks, under experimental obesity and NAFLD induced by a high-fat diet, mice supplemented with EPA or DHA exhibited distinct transcriptomic responses in the liver and muscle. In advanced NAFLD (steatohepatitis associated with fibrosis marks), the DHA reduced genes encoding liver fibrosis and immune cell recruitment, while EPA increased genes associated with cell cycle renew, without affecting the DHA targets [72]. In steatotic Zucker rats (genetic model for obesity and comorbidities) supplemented with EPA or DHA for 8 weeks, both fatty acids were useful through different signaling pathways. The EPA reduced the hepatic gluconeogenesis by inhibiting the FoxO1 transcription factor into the nucleus, while DHA reduced the fatty acid synthase (FAS) enzyme independent of FoxO1 modulation [73]. Despite distinct EPA and DHA actions, the synergy between the two appears to be most effective for global health.^73^ Here, we chose the capsule containing a natural blend similar to what is found in fishes from cold water (Table 1).

Undisputedly, the wrong placebo selection is one of the most significant limitations to pair the comparisons. Oils such as corn, sunflower, and safflower are exuberant sources of linoleic acid (ω6–C18:2), without or with insignificant ω3 (ALA–C18:3) content [47]. Despite ω6 being an essential fatty acid, its excess could induce inflammation due to high prostaglandins (PGE_2_, PGI_2_, TXA_2_) bioconversion [74, 75]. The relevance of ω6:ω3 ratio is currently questionable; however, it is still recommended to maintain this proportion around 5:1 (ω6:ω3). In western or highly industrialized societies, 20:1 is pointed as a risk to diseases with an inflammatory background [76]. Then, the use of ω6 sources as a placebo could overestimate the possible ω3 benefits [67, 77]. On the other hand, olive oil has a broad spectrum of remarkable actions due to high oleic fatty acid (C18:1–ω9) and phenolic compounds [78], underestimating the ω3 benefits. It is not an easy decision; however, molecular investigations have been contributing to reducing the probability of errors associated with fatty acid receptor homology.

The GPR40 is the receptor used by oleic fatty acid to control the intracellular inflammation cascade, similar to ω3, blocking TAK1 phosphorylation [79]; however, in humans, GPR40 has 10% of homology with GPR120 [80]. These receptors are promiscuous for both oleic and ω3 fatty acids, causing misinterpretation through cross-talk signaling [79]. After oleic cell entrance, it binds and activates the PGC1-α (PPAR-γ Co-activator 1-alpha) protein, which induces the nuclear translocation of c-MAF protein, a transcriptional factor responsible for interleukin-10 (IL10) gene transcription, the most potent anti-inflammatory interleukin [81]. Thus, through distinct manners, olive oil can mask the possible ω3 benefits [30, 34, 66]. Here, the mineral oil will be adopted because it has a low probability of interfering in the proposed outcomes or digestive emollient action at this dose [82].

GPR120 is the main ω3 receptor [17] present in several tissues [79], respondings rapidly when this molecule is present. Experimentally, 2, 3, or 4 h after administration of a single dose of a rich ω3 source (flaxseed oil), the GPR120 pathway was activated in the liver, muscle, adipose tissue [79], aorta [42], and retina [41] of obese and insulin-resistant mice. In comparison to the placebo, the ω3-treated group had the βarr2 attracted to GPR120. So far, this approach has not been tested in humans and could reflect the acute action of ω3 fatty acids, and its undescribed antiinflammatory detailed mechanism. We do not expect a reduction in inflammatory markers acutely (after 3 or 4 h); however, if GPR120 will bind to βarr2, the existence of this possibility will be firstly evidenced in humans. The choice for peripheral blood mononuclear cells (PBMCs) was supported by two studies that identified the presence of GPR120 on macrophage surface infiltered in mice aorta [42] and on Kupffer cells in the liver [83]. Raptis et al. (2014) showed the ω3 anti-inflammatory action through TAK1 inhibition, mediated by βarr2 connected to GPR120, which changed the Kupffer polarization from M1 to M2 phenotype, mitigating inflammatory stress in the liver [83].

After acute ω3 testing, subjects will be supplemented with ω3 or placebo for 4 weeks, in accordance to Jiménez-Gómez et al. (2009) [84] and Ras et al. (2014) [85], who respectively showed the reduction on IL6 mRNA content in PBMCs or reduction on hypertriglyceridemic and hypercholesterolemic patients treated with ω3 in capsules during 4 weeks. Not distant, the ER-stress response is intimately connected to inflammatory signaling, counterbalance inflammation, and apoptosis [4, 13, 42]. In hepatocytes, ER stress has a remarkable capacity to adapt to extracellular and intracellular changes to ensure that vital hepatic metabolic functions are preserved. However, in humans, numerous disturbances (e.g., hyperlipidemia, inflammation, drugs) can disturb the hepatocyte ER homeostasis contributing to hepatic lipid metabolism dysregulation and liver disease [86]. Mozzini et al. (2014) [87] described a correlation between ER-stress in PBMC and coronary artery disease. In this sense, we decided to test this correlation in NAFLD subjects and its possible reversal after ω3 supplementation.

GPR120 variation, a non-synonymous mutation (p.R270H), has been associated with obesity in humans [36]. To study such mutations/polymorphisms, large sample size is needed. Although this trial has a few numbers of participants to test for mutations, a test of the GPR120 variation will be conducted to guarantee all known aspects that may be associated with the GPR120 functioning.

Thus, considering the lack of conclusive data regarding the ω3 fatty acid in mediating the resolution of inflammation and its consequences through GPR120 in immune cells of overweight/obese and NAFLD individuals, the present study is crucial for the characterization of the signaling mechanisms and molecules that mediate these effects.

### Strengths and limitations of study design

The strengths of this study include the triple blinding (participants, investigators, and statistical analyst), the ω3 and placebo capsules quality being monitored from the factory development to the blood serum bioavailability and ω3 PBMC incorporation. Limitations include patients taking statins, as the pleiotropic beneficial effects of statin could mask the ω3 benefits.

## Supplementary Information

**Additional file 1:.** Standard Protocol Items: Recommendation for Intervention Trials (SPIRIT)

## Data Availability

Not applicable.

## Abbreviations

βarr2: Beta-arrestin 2
ω3: Omega-3
ATF: Activating transcription factor
CHOP: C/EBP homologous protein
DHA: Docosahexaenoic acid
eIF: Eukaryotic initiation factor
EPA: Eicosapentaenoic acid
FFAR: Free fatty acid receptor
GPR120: G-protein-coupled receptor-120
HSP: Heat shock protein
IL: Interleukin
IRE: Inositol-requiring enzyme
JNK: c-JUN N-terminal-kinase
MCP1: Monocytes chemoattractant protein 1
NAFLD: Non-alcoholic fatty liver disease
NLPR3: NOD-, LRR-, and pyrin domain-containing protein 3
PBMC: Peripheral blood mononuclear cells
PPARg: Peroxisome proliferator-activated receptor-gamma
TAB1: Mitogen-activated protein kinase kinase kinase 7-interacting protein 1
TAK: TGFβ-activated kinase1
TLR4: Toll-like receptor-4
TNF: Tumor necrosis factor
UPR: Unfolded protein response
XBP1: X-box binding protein 1

## References

[1] Peter D. Gluckman, Mark Hanson, Paul Zimmet. T. F. Losing the war against obesity: the need for a developmental perspective. *Sci Transl Med*. 2011;**3**(93):1–4. 10.1126/scitranslmed.3002554. 10.1126/scitranslmed.3002554 21795585

[2] Amor I. Ben *et al*. Midterm outcomes of gastric pouch resizing for weight regain after roux-en-Y gastric bypass. *Obes. Surg.* 2020;**30**(7):2723–8. 10.1007/s11695-020-04560-x. 10.1007/s11695-020-04560-x 32356094

[3] Van De Sande-Lee S (2011). Partial reversibility of hypothalamic dysfunction and changes in brain activity after body mass reduction in obese subjects. Diabetes.

[4] Cintra DE, Ropelle ER, Moraes JC, Pauli JR, Morari J, de Souza CT, Grimaldi R, Stahl M, Carvalheira JB, Saad MJ, Velloso LA (2012). Unsaturated fatty acids revert diet-induced hypothalamic inflammation in obesity. PLoS One.

[5] Thaler JP, Yi CX, Schur EA, Guyenet SJ, Hwang BH, Dietrich MO, Zhao X, Sarruf DA, Izgur V, Maravilla KR, Nguyen HT, Fischer JD, Matsen ME, Wisse BE, Morton GJ, Horvath TL, Baskin DG, Tschöp MH, Schwartz MW (2012). Obesity is associated with hypothalamic injury in rodents and humans. J. Clin. Invest..

[6] Smith GI, Shankaran M, Yoshino M, Schweitzer GG, Chondronikola M, Beals JW, Okunade AL, Patterson BW, Nyangau E, Field T, Sirlin CB, Talukdar S, Hellerstein MK, Klein S (2020). Insulin resistance drives hepatic de novo lipogenesis in nonalcoholic fatty liver disease. J. Clin. Invest..

[7] Odegaard, J. I. & Chawla, A. Pleiotropic actions of insulin resistance and inflammation in metabolic homeostasis. *Science (80-. ).* 339, 172–177 (2013). 10.1126/science.1230721 PMC3725457 23307735

[8] Catrysse L, van Loo G (2017). Inflammation and the metabolic syndrome: the tissue-specific functions of NF-κB. Trends Cell Biol..

[9] Hernández EA, Kahl S, Seelig A, Begovatz P, Irmler M, Kupriyanova Y, Nowotny B, Nowotny P, Herder C, Barosa C, Carvalho F, Rozman J, Neschen S, Jones JG, Beckers J, de Angelis MH, Roden M (2017). Acute dietary fat intake initiates alterations in energy metabolism and insulin resistance. J. Clin. Invest..

[10] Kado A, Tsutsumi T, Enooku K, Fujinaga H, Ikeuchi K, Okushin K, Moriya K, Yotsuyanagi H, Koike K (2019). Noninvasive diagnostic criteria for nonalcoholic steatohepatitis based on gene expression levels in peripheral blood mononuclear cells. J. Gastroenterol..

[11] Myhrstad MCW, Ulven SM, Günther CC, Ottestad I, Holden M, Ryeng E, Borge GI, Kohler A, Brønner KW, Thoresen M, Holven KB (2014). Fish oil supplementation induces expression of genes related to cell cycle, endoplasmic reticulum stress and apoptosis in peripheral blood mononuclear cells: a transcriptomic approach. J. Intern. Med..

[12] Vedin I, Cederholm T, Freund-Levi Y, Basun H, Garlind A, Irving GF, Eriksdotter-Jönhagen M, Wahlund LO, Dahlman I, Palmblad J (2012). Effects of DHA- rich n-3 fatty acid supplementation on gene expression in blood mononuclear leukocytes: the omegAD study. PLoS One.

[13] Hotamisligil GS (2017). Inflammation, metaflammation and immunometabolic disorders. Nature.

[14] Reilly SM, Saltiel AR (2017). Adapting to obesity with adipose tissue inflammation. Nat. Rev. Endocrinol..

[15] H, A. Elevated serum TNF- α is related to obesity in type 2 diabetes mellitus and is associated with glycemic control and insulin resistance. *J. Obes.* 2020, (2020). 10.1155/2020/5076858 PMC7013317 32089876

[16] Shi G, Maminishkis A, Banzon T, Jalickee S, Li R, Hammer J, Miller SS (2008). Control of chemokine gradients by the retinal pigment epithelium. Invest. Ophthalmol. Vis. Sci..

[17] Oh DY, Talukdar S, Bae EJ, Imamura T, Morinaga H, Fan WQ, Li P, Lu WJ, Watkins SM, Olefsky JM (2010). GPR120 is an Omega-3 fatty acid receptor mediating potent anti-inflammatory and insulin-sensitizing effects. Cell.

[18] Zayed EA, AinShoka AA, El Shazly KA, Abd El Latif HA. Improvement of insulin resistance via increase of GLUT4 and PPARγ in metabolic syndrome-induced rats treated with omega-3 fatty acid or l-carnitine. J. Biochem. Mol. Toxicol. 2018;32. 10.1002/jbt.22218 30256492

[19] Daniel H (2016). Hwanga, Jeong-A. Kimb, J. Y. L. Mechanisms for the activation of Toll-like receptor 2/4 by saturated fatty acids and inhibition by docosahexaenoic acid. Eur J Pharmacol.

[20] Huang F, del-Río-Navarro BE, Leija-Martinez J, Torres-Alcantara S, Ruiz-Bedolla E, Hernández-Cadena L, Barraza-Villarreal A, Romero-Nava R, Sanchéz-Muñoz F, Villafaña S, Marchat LA, Hong E (2019). Effect of omega-3 fatty acids supplementation combined with lifestyle intervention on adipokines and biomarkers of endothelial dysfunction in obese adolescents with hypertriglyceridemia. J. Nutr. Biochem..

[21] Bhatt DL, Steg PG, Miller M, Brinton EA, Jacobson TA, Ketchum SB, Doyle RT, Juliano RA, Jiao L, Granowitz C, Tardif JC, Ballantyne CM (2019). Cardiovascular risk reduction with icosapent ethyl for hypertriglyceridemia. N. Engl. J. Med..

[22] Albracht-Schulte K, Kalupahana NS, Ramalingam L, Wang S, Rahman SM, Robert-McComb J, Moustaid-Moussa N (2018). Omega-3 fatty acids in obesity and metabolic syndrome: a mechanistic update. J. Nutr. Biochem..

[23] Dyerberg J, Bang HO, Stoffersen E, Moncada S, Vane JR (1978). Eicosapentaenoic acid and prevention of thrombosis and atherosclerosis? *Lancet (London, England)***2**, 117–9.

[24] Dyerberg J, Bang HO (1979). Haemostatic function and platelet polyunsaturated fatty acids in Eskimos. Lancet (London, England).

[25] Hirasawa A, Tsumaya K, Awaji T, Katsuma S, Adachi T, Yamada M, Sugimoto Y, Miyazaki S, Tsujimoto G (2005). Free fatty acids regulate gut incretin glucagon-like peptide-1 secretion through GPR120. Nat. Med..

[26] Yan Y, Jiang W, Spinetti T, Tardivel A, Castillo R, Bourquin C, Guarda G, Tian Z, Tschopp J, Zhou R (2013). Omega-3 fatty acids prevent inflammation and metabolic disorder through inhibition of NLRP3 inflammasome activation. Immunity.

[27] Duvall MG, Levy BD (1998). Airway inflammation. Chest.

[28] Charles N (2015). Serhan, Jesmond Dalli, Romain A. Colas, Jeremy W. Winkler, and N. C. Protectins and maresins: new pro-resolving families of mediators in acute inflammation and resolution bioactive metabolome. Biochim Biophys Acta.

[29] Manson JAE (2019). Marine n-3 fatty acids and prevention of cardiovascular disease and cancer. N. Engl. J. Med..

[30] Bowman L (2018). Effects of n-3 fatty acid supplements in diabetes mellitus. N. Engl. J. Med..

[31] Arnett DK, Blumenthal RS, Albert MA, Buroker AB, Goldberger ZD, Hahn EJ, Himmelfarb CD, Khera A, Lloyd-Jones D, McEvoy JW, Michos ED, Miedema MD, Muñoz D, Smith SC, Virani SS, Williams KA, Yeboah J, Ziaeian B (2019). 2019 ACC/AHA Guideline on the primary prevention of cardiovascular disease: executive summary. J. Am. Coll. Cardiol..

[32] Santos RD, Gagliardi AC, Xavier HT, Magnoni CD, Cassani R, Lottenberg AM, Arpadi Faludi A, Geloneze B, Scherr C, Kovacs C, Tomazzela C, Carla C, Barrera-Arellano D, Cintra D, Quintão E, Nakandakare ER, Fonseca FA, Pimentel I, Ernesto dos Santos J, Bertolami MC, Rogero M, Izar MC, Nakasato M, Teixeira Damasceno NR, Maranhão R, Cassani RS, Perim R, Ramos S, Sociedade Brasileira de Cardiologia, Sociedade Brasileira de Cardiologia (2013). First guidelines on fat consumption and cardiovascular health. Arq. Bras. Cardiol..

[33] Araujo P, Tilahun E, Zeng Y (2018). A novel strategy for discriminating marine oils by using the positional distribution (sn-1, sn-2, sn-3) of omega-3 polyunsaturated fatty acids in triacylglycerols. Talanta.

[34] Kastelein JJP, Maki KC, Susekov A, Ezhov M, Nordestgaard BG, Machielse BN, Kling D, Davidson MH (2014). Omega-3 free fatty acids for the treatment of severe hypertriglyceridemia: the EpanoVa for lowering very high triglyceridEs (EVOLVE) trial. J. Clin. Lipidol..

[35] Gerling CJ, Whitfield J, Mukai K, Spriet LL (2014). Variable effects of 12 weeks of omega-3 supplementation on resting skeletal muscle metabolism. Appl. Physiol. Nutr. Metab..

[36] Ichimura A, Hirasawa A, Poulain-Godefroy O, Bonnefond A, Hara T, Yengo L, Kimura I, Leloire A, Liu N, Iida K, Choquet H, Besnard P, Lecoeur C, Vivequin S, Ayukawa K, Takeuchi M, Ozawa K, Tauber M, Maffeis C, Morandi A, Buzzetti R, Elliott P, Pouta A, Jarvelin MR, Körner A, Kiess W, Pigeyre M, Caiazzo R, van Hul W, van Gaal L, Horber F, Balkau B, Lévy-Marchal C, Rouskas K, Kouvatsi A, Hebebrand J, Hinney A, Scherag A, Pattou F, Meyre D, Koshimizu TA, Wolowczuk I, Tsujimoto G, Froguel P (2012). Dysfunction of lipid sensor GPR120 leads to obesity in both mouse and human. Nature.

[37] Chan AW, Tetzlaff JM, Altman DG, Laupacis A, Gøtzsche PC, Krleža-Jerić K, Hróbjartsson A, Mann H, Dickersin K, Berlin JA, Doré CJ, Parulekar WR, Summerskill WSM, Groves T, Schulz KF, Sox HC, Rockhold FW, Rennie D, Moher D (2013). SPIRIT 2013 statement: defining standard protocol items for clinical trials. Annals of Internal Medicine.

[38] Organization, W. H WHO_NUT_NCD_98.1_(p1-158).pdf. 275 (1997).

[39] Huang S-S, He S-L, Zhang Y-M (2016). The effects of telmisartan on the nuclear factor of activated T lymphocytes signalling pathway in hypertensive patients. J. Renin. Angiotensin. Aldosterone. Syst..

[40] da Saúde M. *Vigitel Brasil 2018: Vigilância de fatores de risco e proteção para doenças crônicas por inquerito telefônico*. *G. Estatística e Informação em Saúde*. 2019.

[41] Dátilo, M. N. *et al.* Omega-3 from flaxseed oil protects obese mice against diabetic retinopathy through GPR120 receptor. 1–13 (2018). doi:10.1038/s41598-018-32553-5 10.1038/s41598-018-32553-5 PMC6156233 30254287

[42] Moura-Assis A, Afonso MS, de Oliveira V, Morari J, dos Santos GA, Koike M, Lottenberg AM, Ramos Catharino R, Velloso LA, Sanchez Ramos da Silva A, de Moura LP, Ropelle ER, Pauli JR, Cintra DEC (2018). Flaxseed oil rich in Omega-3 protects aorta against inflammation and endoplasmic reticulum stress partially mediated by GPR120 receptor in obese, diabetic and dyslipidemic mice models. J. Nutr. Biochem..

[43] NHANES. Anthropometry procedures manual. *Natl. Heal. Nutr. examinatory Surv.* 1–102 (2007).

[44] Sarry El Din, A., Hassan, N., El-Masry, S. & Al-Tohamy, M. Neck circumference as a simple screening measure for identifying Egyptian overweight and obese adults. Maced. J. Med. Sci*.* 6, 232–237 (2013).

[45] Ashwell M, Hsieh SD (2005). Six reasons why the waist-to-height ratio is a rapid and effective global indicator for health risks of obesity and how its use could simplify the international public health message on obesity. Int. J. Food Sci. Nutr..

[46] Durnin, B. Y. J. V. G. a & Womersley, J. Body fat assessed from total body density and its estimation from skinfold thickness: measurements on 481 men and womwn aged from 16 to 72 years. *Br. J. Nutr.* 32, 77–97 (1973). 10.1079/bjn19740060 4843734

[47] NEPA. TACO. *Public domain* 164 (2011). Available at: http://www.nepa.unicamp.br/taco/tabela.php?ativo=tabela. (Accessed: 18th June 2020)

[48] IBGE. Brazilian Institute of Geography and Statistics. Demographic Census. (2010). Available at: https://censo2010.ibge.gov.br/. (Accessed: 7th September 2020)

[49] Nusser SM, Carriquiry AL, Dodd KW, Fuller WA (1996). A semiparametric transformation approach to estimating usual daily intake distributions. J. Am. Stat. Assoc..

[50] Willett W, Stampfer MJ (1986). Total energy intake: implications for epidemiologic analyses. Am. J. Epidemiol..

[51] Matsudo S (2012). Questionário Internacional De Atividade Física (Ipaq): Estupo De Validade E Reprodutibilidade No Brasil. Rev. Bras. Atividade Física Saúde.

[52] Krogulska A, Borowiec M, Polakowska E, Dynowski J, Młynarski W, Wasowska-Królikowska K (2011). FOXP3, IL-10, and TGF-β genes expression in children with IgE-dependent food allergy. J. Clin. Immunol..

[53] Muñoz VR, Gaspar RC, Kuga GK, da Rocha AL, Crisol BM, Botezelli JD, Baptista IL, Mekary RA, da Silva ASR, Cintra DE, de Moura LP, Ropelle ER, Pauli JR (2018). Exercise increases Rho-kinase activity and insulin signaling in skeletal muscle. J. Cell. Physiol..

[54] Hartman, L. & Lago, R. C. Rapid preparation of fatty acid methyl esters from lipids. Lab. Pract*.* 22, 475–476 passim (1973). 4727126

[55] Shirai N, Suzuki H, Wada S (2005). Direct methylation from mouse plasma and from liver and brain homogenates. Anal. Biochem..

[56] McCoy CE (2017). Understanding the intention-to-treat principle in randomized controlled trials. Western Journal of Emergency Medicine.

[57] Wang DD, Hu FB (2017). Dietary fat and risk of cardiovascular disease: recent controversies and advances. Annu. Rev. Nutr..

[58] De Mello VDF (2009). The effect of fatty or lean fish intake on inflammatory gene expression in peripheral blood mononuclear cells of patients with coronary heart disease. Eur. J. Nutr..

[59] Betancor MB, et al. An oil containing EPA and DHA from transgenic Camelina sativa to replace marine fish oil in feeds for Atlantic salmon (Salmo salar L.): effects on intestinal transcriptome, histology, tissue fatty acid profiles and plasma biochemistry. PLoS One. 2017;12. 10.1371/journal.pone.0175415 PMC5389825 28403232

[60] Cintra DEC, Costa AGV, Peluzio MCG, Matta SLP, Silva MTC, Costa NMB (2006). Lipid profile of rats fed high-fat diets based on flaxseed, peanut, trout, or chicken skin. Nutrition.

[61] Satué MT, López MC (1996). Sex-linked differences in fatty acid composition of rainbow trout (Oncorhynchus mykiss) liver oil. Food Chem..

[62] Ribeiro CS, Gomes AD, Vieira VARO, Tabata YA, Takahashi NS, Moreira RG (2012). The effect of ploidy on the fatty acid profile during the reproductive cycle of female rainbow trout (Oncorhynchus mykiss). Aquac. Int..

[63] Ritter JCS, Budge SM, Jovica F (2013). Quality analysis of commercial fish oil preparations. J. Sci. Food Agric..

[64] Galuch M (2017). Quality assessment of Omega-3 supplements available in the Brazilian market. J. Braz. Chem. Soc..

[65] Gruppo GI (1999). Dietary supplementation with n-3 polyunsaturated fatty acids and vitamin E after myocardial infarction: results of the GISSI-Prevenzione trial. Gruppo Italiano per lo Studio della Sopravvivenza nell’Infarto miocardico. Lancet (London, England).

[66] Hande LN, Thunhaug H, Enebakk T, Ludviksen J, Pettersen K, Hovland A, Lappegård KT (2019). Addition of marine omega-3 fatty acids to statins in familial hypercholesterolemia does not affect in vivo or in vitro endothelial function. J. Clin. Lipidol..

[67] Skulas-Ray AC, Kris-Etherton PM, Harris WS, West SG (2012). Effects of marine-derived omega-3 fatty acids on systemic hemodynamics at rest and during stress: a dose-response study. Ann. Behav. Med..

[68] Nodari S, Triggiani M, Campia U, Manerba A, Milesi G, Cesana BM, Gheorghiade M, Dei Cas L (2011). Effects of n-3 polyunsaturated fatty acids on left ventricular function and functional capacity in patients with dilated cardiomyopathy. J. Am. Coll. Cardiol..

[69] Klingel, SL; Metherel, AH; Irfan, M; Rajna, A; Chabowski, A; Bazinet, RP; Mutch, D. EPA and DHA have divergent effects on serum triglycerides and lipogenesis, but similar effects on lipoprotein lipase activity: a randomized controlled trial - PubMed. 1502–1509 (2019). 10.1093/ajcn/nqz234 31535138

[70] Vors C, Allaire J, Marin J, Lépine MC, Charest A, Tchernof A, et al. Inflammatory gene expression in whole blood cells after EPA vs. DHA supplementation: results from the ComparED study. *Atherosclerosis*. 2017;257:116–22. 10.1016/j.atherosclerosis.2017.01.025. 10.1016/j.atherosclerosis.2017.01.025 28131045

[71] Mozaffarian D, Wu JHY. (n-3) Fatty acids and cardiovascular health: are effects of EPA and DHA shared or complementary? J. Nutr. 2012;142. 10.3945/jn.111.149633 PMC3278271 22279134

[72] Kunz HE, Dasari S, Lanza IR (2019). EPA and DHA elicit distinct transcriptional responses to high-fat feeding in skeletal muscle and liver. Am. J. Physiol. Endocrinol. Metab..

[73] Hong L, Zahradka P, Cordero-Monroy L, Wright B, Taylor CG. Dietary docosahexaenoic acid (DHA) and eicosapentaenoic acid (EPA) operate by different mechanisms to modulate hepatic steatosis and hyperinsulemia in fa/fa Zucker rats. *Nutrients*. 2019;11(4). 10.3390/nu11040917. 10.3390/nu11040917 PMC6521162 31022865

[74] Kain V, Ingle KA, Kachman M, Baum H, Shanmugam G, Rajasekaran NS, Young ME, Halade GV (2018). Excess ω-6 fatty acids influx in aging drives metabolic dysregulation, electrocardiographic alterations, and low-grade chronic inflammation. Am. J. Physiol. - Hear. Circ. Physiol..

[75] Rallidis, L. S. *et al.* Dietary a -linolenic acid decreases C-reacti v e protein , serum amyloid A and interleukin-6 in dyslipidaemic patients. 167, (2003). 10.1016/s0021-9150(02)00427-6 12818406

[76] Simopoulos AP (2016). An increase in the Omega-6/Omega-3 fatty acid ratio increases the risk for obesity. Nutrients.

[77] Rodríguez-Cruz M, Cruz-Guzmán OR, Almeida-Becerril T, Solís-Serna AD, Atilano-Miguel S, Sánchez-González JR, Barbosa-Cortés L, Ruíz-Cruz ED, Huicochea JC, Cárdenas-Conejo A, Escobar-Cedillo RE, Yam-Ontiveros CA, Ricárdez-Marcial EF (2018). Potential therapeutic impact of omega-3 long chain-polyunsaturated fatty acids on inflammation markers in Duchenne muscular dystrophy: a double-blind, controlled randomized trial. Clin. Nutr..

[78] Estruch R (2018). Primary prevention of cardiovascular disease with a mediterranean diet supplemented with extra-virgin olive oil or nuts. N. Engl. J. Med..

[79] Oliveira V, Marinho R, Vitorino D, Santos GA, Moraes JC, Dragano N, Sartori-Cintra A, Pereira L, Catharino RR, da Silva ASR, Ropelle ER, Pauli JR, de Souza CT, Velloso LA, Cintra DE (2015). Diets containing α-Linolenic (ω3) or Oleic (ω9) fatty acids rescues obese mice from insulin resistance. Endocrinology.

[80] Hirasawa A, Hara T, Katsuma S, Adachi T, Tsujimoto G (2008). Free fatty acid receptors and drug discovery. Biol. Pharm. Bull..

[81] Morari J, Torsoni AS, Anhê GF, Roman EA, Cintra DE, Ward LS, Bordin S, Velloso LA (2010). The role of proliferator-activated receptor γ coactivator-1α in the fatty-acid-dependent transcriptional control of interleukin-10 in hepatic cells of rodents. Metabolism..

[82] Nogueira MA, Oliveira CP, Ferreira Alves VA, Stefano JT, Rodrigues LSR, Torrinhas RS, Cogliati B, Barbeiro H, Carrilho FJ, Waitzberg DL (2016). Omega-3 polyunsaturated fatty acids in treating non-alcoholic steatohepatitis: a randomized, double-blind, placebo-controlled trial. Clin. Nutr..

[83] Raptis DA, Limani P, Jang JH, Ungethüm U, Tschuor C, Graf R, Humar B, Clavien PA (2014). GPR120 on Kupffer cells mediates hepatoprotective effects of ω3-fatty acids. J. Hepatol..

[84] Jiménez-Gómez Y, López-Miranda J, Blanco-Colio LM, Marín C, Pérez-Martínez P, Ruano J, Paniagua JA, Rodríguez F, Egido J, Pérez-Jiménez F (2009). Olive oil and walnut breakfasts reduce the postprandial inflammatory response in mononuclear cells compared with a butter breakfast in healthy men. Atherosclerosis.

[85] Ras RT, Demonty I, Zebregs YEMP, Quadt JFA, Olsson J, Trautwein EA (2014). Low doses of eicosapentaenoic acid and docosahexaenoic acid from fish oil dose- dependently decrease serum triglyceride concentrations in the presence of plant sterols in hypercholesterolemic men and women. J. Nutr..

[86] Lebeaupin C, Vallée D, Hazari Y, Hetz C, Chevet E, Bailly-Maitre B (2018). Endoplasmic reticulum stress signalling and the pathogenesis of non-alcoholic fatty liver disease. Journal of Hepatology.

[87] Mozzini C, Fratta Pasini A, Garbin U, Stranieri C, Pasini A, Vallerio P, Cominacini L (2014). Increased endoplasmic reticulum stress and Nrf2 repression in peripheral blood mononuclear cells of patients with stable coronary artery disease. Free Radic. Biol. Med..

